# A Common Stem Cell for Murine Cortical and Medullary Thymic Epithelial Cells?

**DOI:** 10.1155/1995/23168

**Published:** 1995

**Authors:** Carsten Röpke, Peter Van Soest, Peter Paul Platenburg, Willem Van Ewijk

**Affiliations:** 1 Department of Medical Anatomy University of Copenhagen the Panum Institute Blegdamsvej 3 Copenhagen N DK-2200 Denmark; 2 Department of Immunology Erasmus University Rotterdam 3000 DR The Netherlands

**Keywords:** Fetal cell culture, fetal murine thymus, stem cell, thymic epithelium

## Abstract

We have addressed the question whether the epithelial stroma in the thymus is derived from a common stem cell or whether cortical and medullary epithelial cells are derived from different embryonic stem cells emerging, for example, from endoderm and ectoderm. By the use of rapidly expanding cultures of thymic epithelial cells (TEC) from 14 to 16 day-old murine fetuses and by specific antibodies against cortical and medullary epithelium, respectively, we were able to demonstrate a small subpopulation of double-labeled TEC in the cultures. These cells were not present in TEC cultures initiated from thymuses of neonatal mice. Double-labeled TEC were also found in tissue sections from fetal thymuses. These findings may indicate that TEC populations of the cortex and the medulla are derived from a common stem cell, with potential for differentiation toward both cortical and medullary TEC.


*Developmental Immunology*, 1995, Vol 4, pp. 149–156
Reprints available directly from the publisher
Photocopying permitted by license only

© 1995 Harwood Academic Publishers GmbH
Printed in Singapore

### A Common Stem Cell for Murine Cortical and Medullary Thymic Epithelial Cells?

CARSTEN RÖPKE,*† PETER VAN SOEST,‡ PETER PAUL PLATENBURG,‡ and WILLEM VAN EWIJK‡

†*Department of Medical Anatomy, University of Copenhagen, the Panum Institute, Blegdamsvej 3, DK-2200 Copenhagen N, Denmark*
‡*Department of Immunology, Erasmus University, 3000 DR Rotterdam, The Netherlands*

We have addressed the question whether the epithelial stroma in the thymus is derived from a common stem cell or whether cortical and medullary epithelial cells are derived from different embryonic stem cells emerging, for example, from endoderm and ectoderm. By the use of rapidly expanding cultures of thymic epithelial cells (TEC) from 14 to 16 day-old murine fetuses and by specific antibodies against cortical and medullary epithelium, respectively, we were able to demonstrate a small subpopulation of double-labeled TEC in the cultures. These cells were not present in TEC cultures initiated from thymuses of neonatal mice. Double-labeled TEC were also found in tissue sections from fetal thymuses. These findings may indicate that TEC populations of the cortex and the medulla are derived from a common stem cell, with potential for differentiation toward both cortical and medullary TEC.

KEYWORDS: Fetal cell culture, fetal murine thymus, stem cell, thymic epithelium.

### INTRODUCTION

It is generally accepted that the thymus develops from the endoderm of the third pharyngeal pouches and the ectoderm of the corresponding branchial cleft. During ontogeny, this anlage is invaded by mesodermal cells: macrophages, dendritic cells, fibroblasts, and T-cell precursors, as reviewed by Lampert and Ritter (1988), von Gaudecker (1991) and van Ewijk (1991). The notion that thymic epithelial cells (TEC) are derived from both ectoderm and endoderm is based on morphological evidence showing that pouches from the two derms approach each other and stay in close contact for some time (Cordier and Heremans, 1975), and that a normal thymus fails to develop if they do not meet (Cordier and Haumont, 1980). This notion is supported by the use of antibodies specific for subpopulations of TEC and immunohistochemistry. Thus, the general findings are that cortical TEC differs from medullary and subcapsular TEC phenotypically (Lampert and Ritter, 1988). Also functional differences between cortical and medullary TEC in positive and negative selection of thymocytes have been documented (reviewed by Boyd and Hugo, 1991).

However, the morphological findings do not prove that TEC actually are derived from both derms, but merely that both derms are of importance for the initiation of the thymus anlage. Thus, the one derm may act as an inducer and the other as the producer of TEC. Furthermore, phenotypical and functional differences between cortical and medullary TEC may as well be due to microenvironmental differences created by lymphoid cells, as indicated by findings in bone marrow reconstituted SCID mice (Shores et al., 1991) and in cocultures between thymocytes and TEC (Berrih et al., 1985; Singer and Haynes, 1987). Haynes et al. (1984) have demonstrated that all TEC in early human fetal thymuses stain with the same antibody (A2B5), which only later is confined to subcapsular and medullary regions. In the adult human thymus, TEC positive for both cortical and medullary markers have been demonstrated, and such cells have also been shown in thymic tumors (reviewed by Lampert and Ritter, 1988, and by Ritter and Boyd, 1993). These observations support the notion that there may exist a stromal "stem cell" giving rise to both

*Corresponding author. Present address: Laboratory for Cellular Immunology, Department of Medical Anatomy A, University of Copenhagen, the Panum Institute, Blegdamsvej 3, DK-2200 Copenhagen N, Denmark.

medullary and cortical TEC. In the light of the previously mentioned immunochemistry studies showing clear separation between cortical and medullary TEC, it is obvious that candidates for such a stem cell, cells that are labeled with both cortical and medullary markers, are not common in the thymus. Therefore, we found it relevant to seek such cells in a situation where the TEC population was rapidly expanding by cell division. For this, we have cultured TEC obtained from 14 to 16-day-old murine fetuses in a serum-free medium, which only allows for TEC growth, and labeled the cells with specific antibodies against cortical and medullary TEC. The results show consistently that a small but definite number of the cultured TEC bind both antibodies, indicating the presence in these cultures of cells with potentials for differentiation to both cortical and medullary TEC. This notion is supported by parallel findings in tissue sections from fetal thymuses.

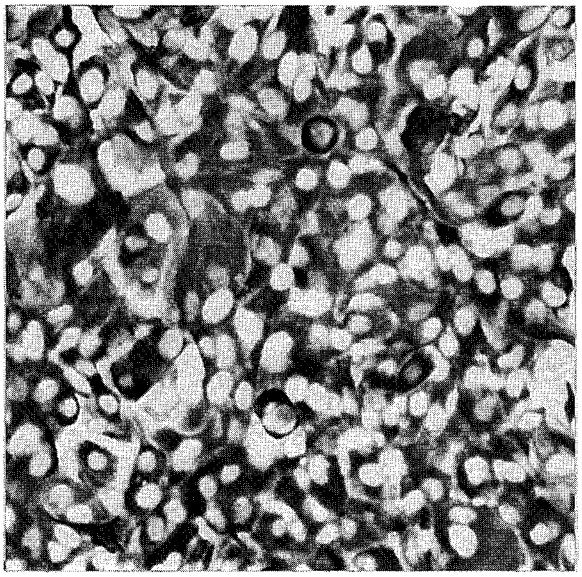

FIGURE 1. Photomicrograph of cultured murine thymus epithelial cells. The cells were derived from 14-day-old fetuses and have been grown for 10 days in serum-free medium. Cells were stained by the PAP method after incubation with antibody against cytokins 6 and 18 (× 252).

### RESULTS

### Characterization of TEC Cultures

Fetal TEC were cultured in a serum-free medium with well-defined growth factors, previously established for culture of TEC from young mice (Röpke et al., 1990). Epithelial cells spread out from the thymic explants within 24 hr, forming a sheet of TEC around the explants. This sheet expanded further in the following 3 weeks used for study. The cell proliferation in these cultures was about double as fast as in cultures of neonatal TEC (data not shown).

By the use of an antibody against cytokeratins (M 717), essentially all cells were found to be labeled (Fig. 1), in agreement with findings in cultures of TEC from young mice (Röpke et al., 1990), indicating an epithelial nature of all cells. Moreover, by the use of the anti-macrophage antibody Mac-1, we were unable to detect cells of the mononuclear phagocyte system (data not shown).

The majority of the cells were of a rather small size—from 10 to 15 μm in diameter—but also considerably larger cells were found. No significant differences in the numbers of cytokeratin-positive cells or in the cell-size distribution were found between cultures established from thymuses of different ages or between cultures of different ages (data not shown).

### Phenotypical Characterization of Fetal TEC

By the use of monoclonal anti-class I and II antibodies, we found that essentially all cells were class I-positive, whereas class II expression was generally weak or absent (data not shown), similar to cultures derived from young mice (Röpke et al., 1990).

With the antibodies ER-TR4 and ER-TR5, specific for cortical and medullary TEC, we consistently found by cell counting that the vast majority of cells (80–90%) were positive for ER-TR5, and ER-TR4 cells were sparse (5–20%), often being situated as a few cells together or occupying small separate areas of 20 to 100 cells in the cell islets. Representative photos of ER-TR4 and ER-TR5-labeled TEC cultures are shown Fig. 2. The previous findings were observed in all cultures irrespective of their ages or of the fetal age of the lobes at the onset of the cultures (data not shown). In order to determine the percentages of TR4 and TR5 cells, TEC from various cultures according to fetal or culture age were detached by trypsination, fixed, and labeled with either TR4- or TR5-FITC. These cell suspensions were then subjected to FACScan analysis. Figure 3 shows typical results: About 40–50% of the cultured TEC are positive for TR5, whereas only 10–15% are TR4-positive.

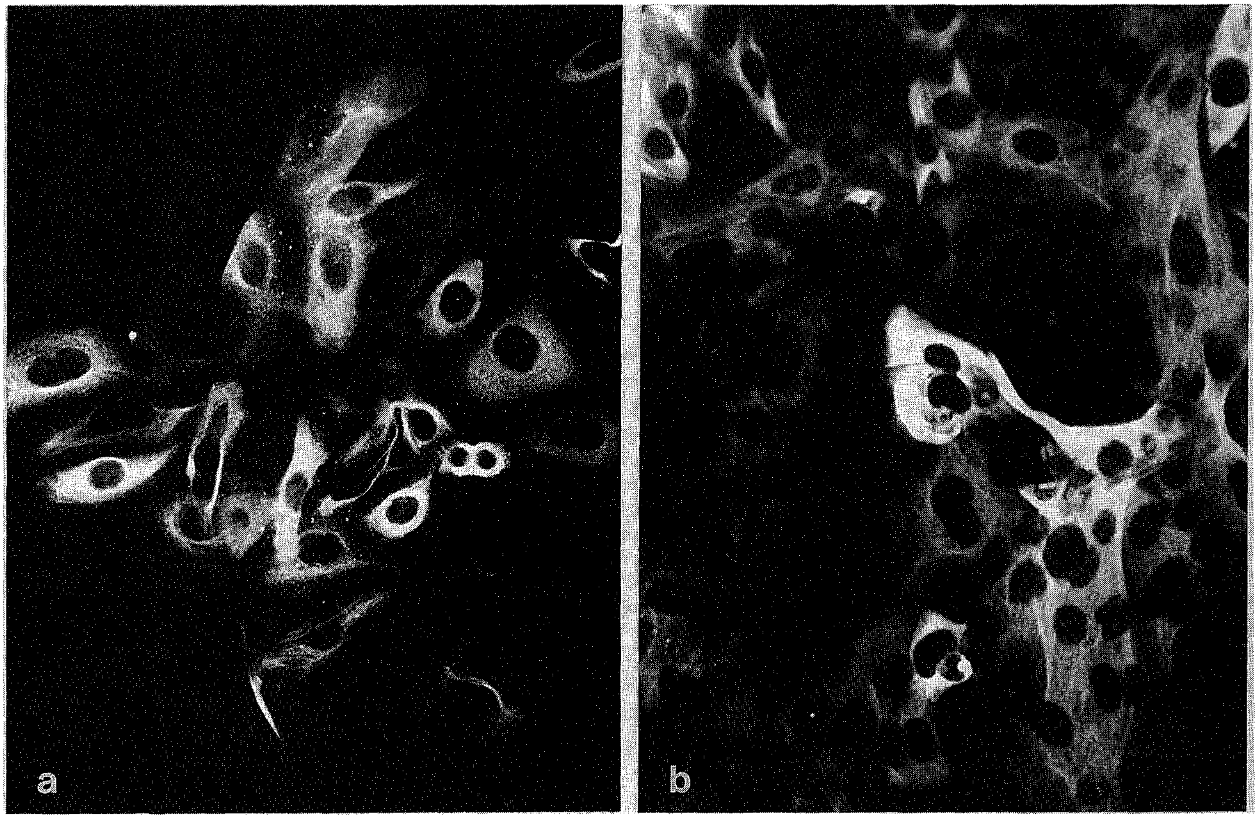

FIGURE 2. Fluorescence photomicrographs of cultured murine thymic epithelial cells. (a) Cells were labeled with ER-TR4 antibody against cortical epithelial cells. (b) Cells were labeled with ER-TR5 antibody against medullary epithelial cells. RaRA-FITC was used as second layer for detection of both antibodies. The cells were derived from 15-day-old fetuses, and were grown for 12 days in serum-free medium (× 250).

To detect stromal stem cells labeled with both TR4 and TR5, a combination of TR4 + RaRA-FITC and TR5-biotin + avidin-Texas red—or the opposite labeling—was employed on the cultures. The results paralleled the previous findings from cultures labeled with only one antibody, irrespective of which of the two antibody combinations was used. However, a limited number of stromal cells labeled with both the cortical and medullary marker was consistently found. These cells constituted always less than 1% of the cells, and they usually occurred as individual cells, or a few together, as shown in Figs. 4(a) to 4(d). As can be seen from the figures, both small and large cells were found to be double-positive, and there was no preferential localization within the cell islets. Furthermore, we were not able to observe changes in numbers of double-labeled cells within the investigated time period of 3 weeks.

### Immunohistological Localization of Double-Labeled Cells in Fetal Thymuses

To investigate the presence of double-labeled TEC *in vivo*, tissue sections were prepared from 14-to-16-day-old fetal thymuses. After incubation with the ER-TR4 and ER-TR5 antibodies, a small number of double-labeled cells were seen in the cortical areas without preferential localization to, for example, the cortico-medullary junction or the subcapsular area (Fig. 5). This observation indicates that double-positive (TR4 +TR5 +) stromal cells occur *in situ*, and that they are not artifacts.

### DISCUSSION

The present results confirm previous results obtained from culture of TEC from newborn mice and

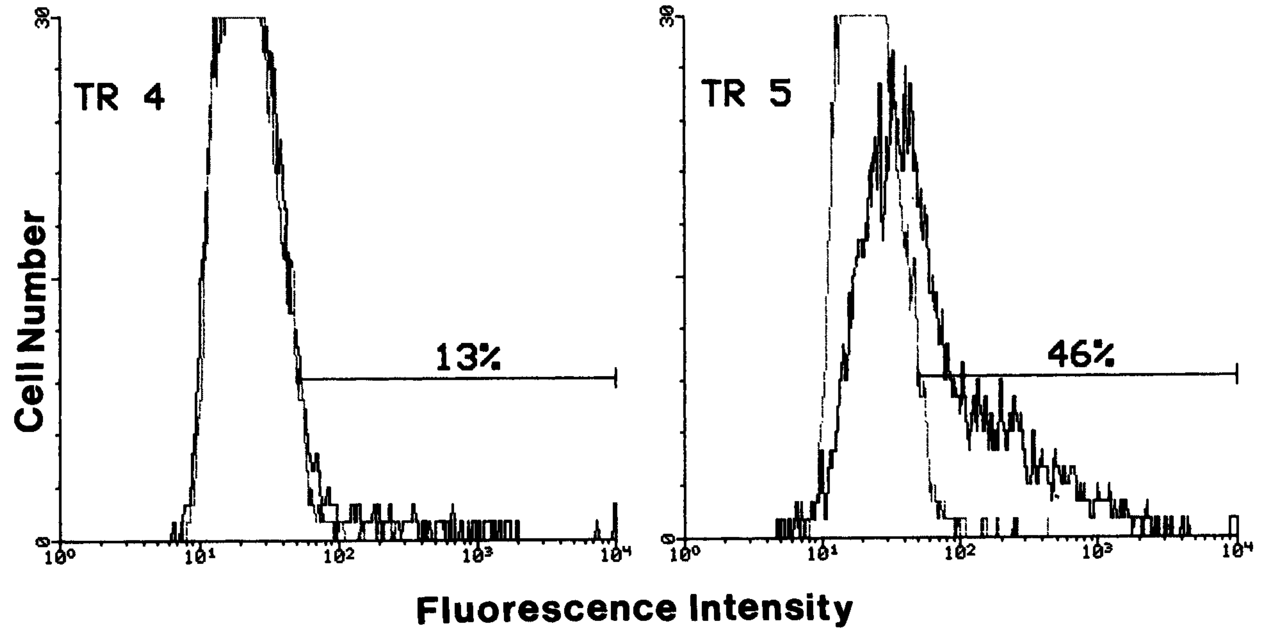

FIGURE 3. Histograms showing results from a typical experiment in which cultured thymic epithelial cells derived from a 15-day-old murine fetus were labeled with either RaRA-FITC (thin line), TR4 + RaRA-FITC for detection of cortical epithelial cells (TR4: heavy line), or TR5 + RaRA-FITC for detection of medullary epithelial cells (TR5: heavy line).

from human thymuses, showing essentially all cultured cells to be of epithelial nature when grown in the present serum-free medium with added, defined growth factors (Röpke et al., 1990; Röpke and Elbroend, 1992). However, in opposition to earlier findings from cultures of TEC obtained from neonatal—or older—thymuses which showed exclusively ER-TR5 +, that is, medullary TEC to constitute the cultures (Röpke et al., 1990), we consistently found cells positive for a cortical TEC marker (TR4) in cultures of fetal TEC beside TEC-positive for the medullary TEC marker (TR5). The results obtained by microscopy were paralleled by FACScan results, although a lower percentage of TR5 + cells was found here. At this moment, we cannot explain why the frequency of TR5 + cells, as determined by FACScan analysis, is lower than the frequency estimated by fluorescence microscopy, but the proportion of TR5 + to TR4 + cells is clearly reflected by these FACScan results.

The main objective of the present study was to verify a possible existence of cells positive for both cortical and medullary TEC markers, that is, cells that may have potentials for development into cortical as well as medullary TEC subsets. Such double-positive cells were present in the fetal cultures from the start of the cultures—or at least after a few days of culture—and maintain their frequency in the cultures, indicating that these cells act as precursors, which give rise to more differentiated TEC of cortical or medullary type. This is at variance with results obtained by Cirne-Lima et al. (1993), who found increasing numbers of double-positive cells in cultures of a TEC line with increasing culture time. In this latter situation, it seems that a subpopulation of cells with potential for development in both cortical and medullary direction is selected, when the cell line is adjusting to the culture situation. In the present fetal cultures, the double-positive TEC are present from the start of the cultures. The maintained proportion in our TEC cultures between TR4- and TR5-positive cells in the culture period adds to the notion that a balance between precursors and more differentiated TEC is obtained in the present culture system. Our results, showing the presence of double-positive TEC in fetal cultures, but not in cultures obtained from neonatal mice (2 to 7 days old), may be explained by the high growth rate in fetal cultures or by an age-related shift in TEC populations concomittant with shifts in thymocyte populations around birth. Because the present cultures are obtained from fetal thymuses that are populated to different extents with thymocytes—$CD4^-CD8^-$ thymocytes at

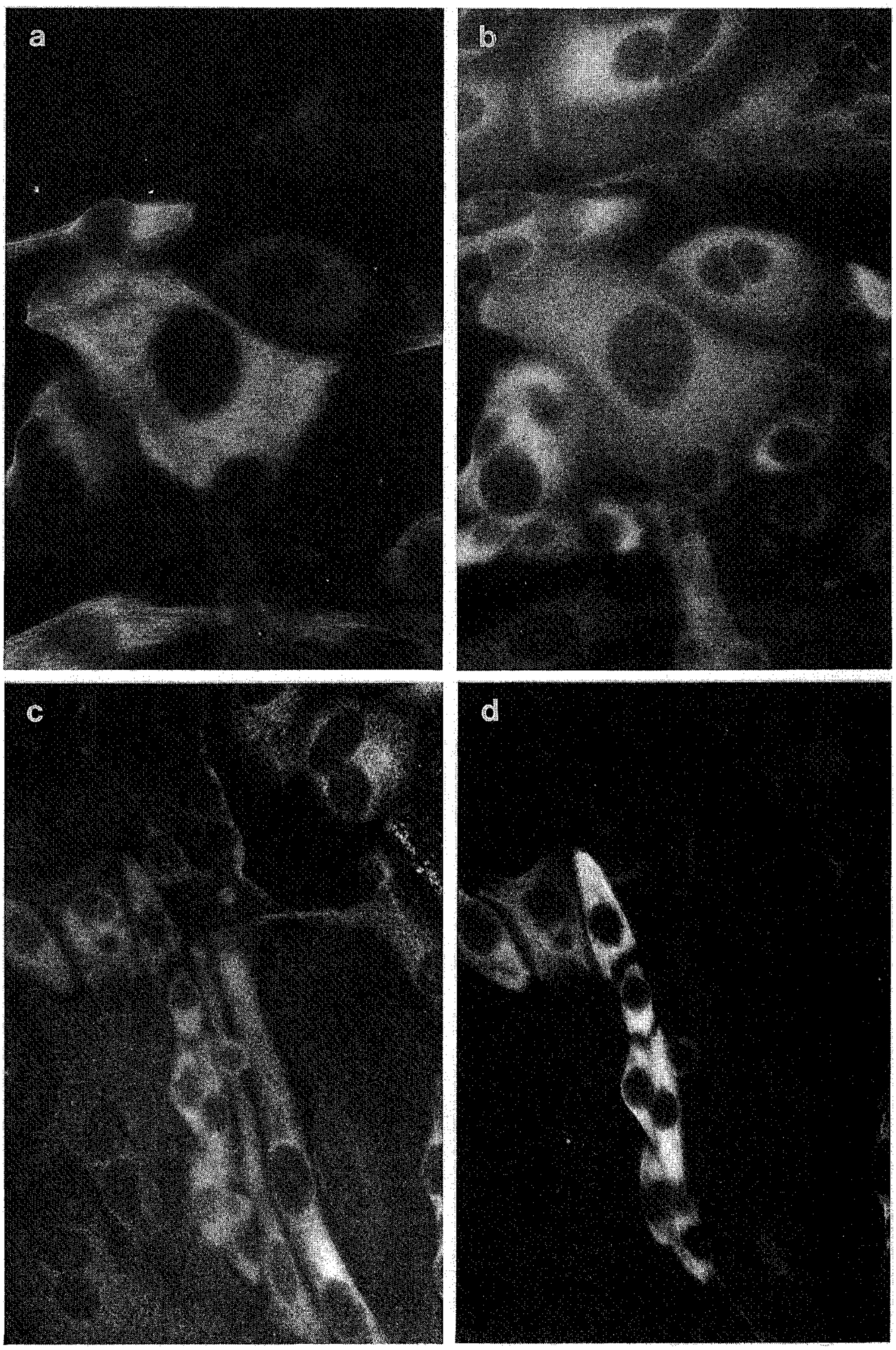

FIGURE 4. Photomicrographs of double-stained cultured murine thymic epithelial cells. Cells were labeled with either biotinylated ER-TR4 + avidin-Texas red (Tex) and ER-TR5 + RaRA-FITC or biotinylated ER-TR5 + avidin-Texas red and ER-TR4 + RaRA-FITC, and photos were taken of the same cells (a and b, or c and d) by alternative use of red and green optic filters (a) TR4-FITC, (b) TR5-Tex (× 916), (c) TR4-Tex, and (d) TR5-FITC (× 458).

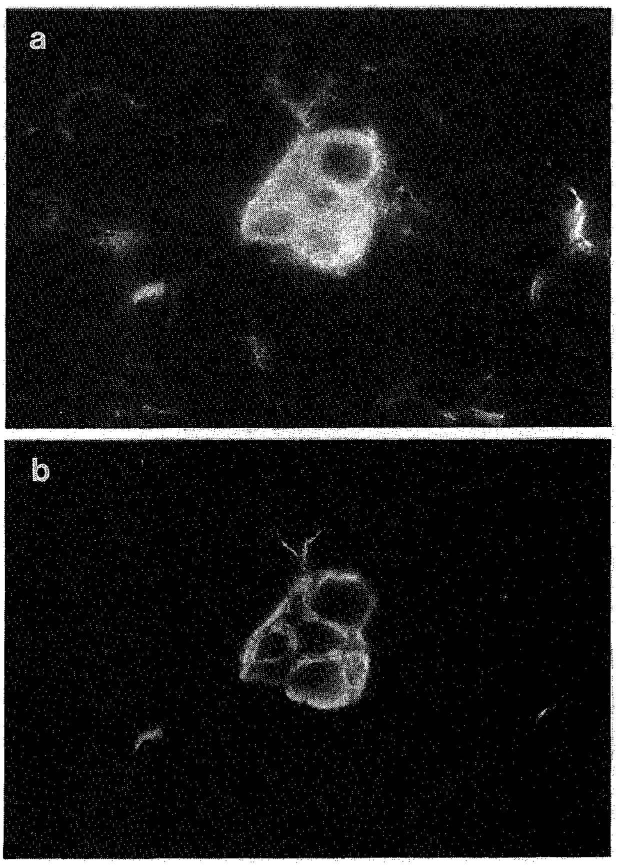

FIGURE 5. Photomicrographs of a section from the thymus of a 17-day-old murine fetus, showing double-labeled cells. (a) Labeling with ER-TR5 + RARA-FITC. (b) Labeling with biotinylated ER-TR4 + avidin-Texas red ( × 458).

day 14–15 of fetal life, and in addition with $CD4^+CD8^+$,$CD4^+$, and $CD8^+$ cells from day 16 (Hugo et al., 1991), it would be of interest to test TEC obtained from thymuses before T-cell precursors invade the tissue.

Recent results obtained after intrathymic injection of TEC line cells of medullary nature, or fibroblasts (Hugo et al., 1992; Vukmanovic et al., 1992; Hugo et al., 1993), indicate that these cells are able to induce positive selection of thymocytes. Besides, cells from a TEC line, which induced positive selection (Hugo et al., 1992), were able to induce negative selection *in vitro* (Hugo et al., 1994). These findings strengthen further the importance of a more detailed knowledge of the thymic micro environments—of the crosstalk between stromal cells and thymocyte subpopulations, and of the plasticity and potential of the stromal cells.

This study demonstrates that epithelial cells with antigens characteristic of cortical and medullary epithelium are confined to the fetus. These cells could have the potential to develop into more differentiated cells with restricted functions and phenotypes. However, the previously mentioned findings in humans (Lampert and Ritter, 1988; Ritter and Boyd, 1993) and in a cell line (Cirne-Lima et al., 1993) may point to the fact that multipotential epithelial cells exist in small numbers in the thymus both before and after birth, and that they become more numerous in situations in which cell proliferation is prominent, for example, in the fetal period, in thymic tumors, and by changes in microenvironment (van Ewijk et al., 1994). We realize that these data do not prove that TR4+TR5+cells are indeed precursors of TR4 and TR5 single positive cells. To verify this, isolation of such cells with the cell sorter and prolonged cell culture is required.

### MATERIALS AND METHODS

### Animals

All embryos were obtained from C3H females, mated with C3H males, which were received from Bomholtgaard, Laven, Denmark, and kept in the animal facilities of our institute. The age of the embryos was determined by scoring the day of appearance of vaginal plugs, which is taken as day 0. Neonatal mice were also of the C3H strain.

### Cell Cultures

Thymic lobes or fragments of these, obtained by gently cutting with surgical knives, were incubated for 1 hr at 37°C in medium containing 5.0 mg/ml DNAase (Sigma, St. Louis, MO) and 1.5 mg/ml collagenase/dispase (Boehringer, Mannhein, Germany) in a culture dish, which was shaken regularly. Thereafter, the sizes of the fragments were considerably below 1 $mm^3$. The lobes and fragments were washed two times with the medium before cultivation.

The serum-free, growth-factor-defined medium used was composed of a 1:1 mixture (v/v) of Ham's F12 medium and Dulbecco's modified Eagle medium (DMEM) (Flow Laboratories, Hillerød, Denmark). The DMEM was prepared without calcium (Medium-Service, Institute of Medical Microbiology, University of Copenhagen, Denmark). The medium was supplemented with 2 mM glutamine (Sigma, G 3126), 250 IU penicillin/ml, 25 mg streptomycine/ml, 3 μg/ml insulin (I, 0.025 IU/mg, Nordisk Insulin, Denmark), 100 ng/ml epidermal growth factor (EGF), and 0.5 μg/ml hydrocortisone (HC) (both from Collaborative Research, USA), and 10 ng/ml cholera toxin (CT Sigma).

The cells were cultured in 1 ml medium in 2-chamber Lab-Tec Chamber slides (NUNC, Roskilde, Denmark). All containers for culture were coated with type I collagen, 8 mg/cm$^2$ (Vitrogen-100; Flow Laboratories, Birkerød, Denmark). Cultures were maintained in a humidified atmosphere of 5% $CO_2$ with 20% $O_2$ and 75% $N_2$. The medium was changed every 2 to 3 days.

### Antibodies

Cytokeratins were detected in a triple-staining method starting with DAKO-CK1 (M 717) specific to multiple keratins (DAKOPATTS, Denmark). The second-stage antibody used was a rabbit polyclonal antibody against mouse immunoglobulins (259, DAKO), and the third reagent was a monoclonal peroxidase anti-peroxidase (PAP) complex (P850, DAKO). The cytochemical incubation was performed as previously described (Petersen and van Deurs, 1988). To detect class I and II MHC antigens on TEC surfaces, an anti-H-2K$^k$ antibody (IgG2a, 16-1-11N, ATCC HB16) (Ozato et al., 1980) and an anti-I-E and -I-A antibody (IgG2b, M5/114.15.2, ATCC TIB120) with specificity for H-2$^b$, H-2$^d$, H-2$^k$, H-2$^q$, and H-2$^r$ (Bhattacharya et al., 1981), were used. The antibodies were applied either directly in the culture chambers or in suspension after trypsination with a trypsin/EDTA solution (Boehringer). No fixative was used. The second-step antibody was a FITC-labeled rabbit-anti-rat IgG antiserum (Nordic, Tilburg, The Netherlands). Mac1-FITC (Boehringer) was used as an anti-macrophage antibody. For detection of murine cortical and medullary TEC, ER-TR4 and ER-TR5 monoclonal antibodies were used. These antibodies were raised by injecting rats with C3H stromal cells, and were shown to label cortical (TR4) and medullary (TR5) TEC subsets in the adult and fetal thymus specifically (Van Vliet et al., 1984, 1985).

### Immunocytochemistry on Stromal Cell Cultures

Chamber slide cultures to be used for labeling with ER-TR4 and ER-TR5 were fixed in methanol for 5 min at −20°C. After drying, slides were treated for 10 min with 10% normal goat serum (NGS) in PBS before addition of ER-TR4, ER-TR5, or PBS. After incubation for 45 min, the slides were rinsed twice with 0.05% Tween in PBS, the slides being placed on a shaking machine for 10 min. After removal of the PBS, the slides were incubated and rinsed as before, the antibody being RaRA-FITC (DAKO, diluted 1:20 in PBS + 10% NGS). Slides were then incubated for 10 min with 2% normal rat serum in PBS, washed, and incubated for 45 min with purified, biotinylated ER-TR4 or ER-TR5 antibodies, rinsed as before, and incubated for another 45 min with avidin-Texas red (Becton Dickinson, Sunnyvale, CA). Finally, slides were rinsed as before and mounted with paraphenylenediamine.

To analyze single cells labeled with TR4 or TR5, TEC cultures were treated with trypsin-EDTA, and TEC in suspension were fixed in methanol for 5 min at −20°C, washed, treated for 10 min with 10% NGS in PBS before addition of ER-TR4, ER-TR5, or PBS. After incubation for 45 min, the cells were washed twice with 0.05% Tween in PBS, and incubated with RaRA-FITC for 30 min, washed, and suspended in PBS for FACScan analysis.

### Immunocytochemistry on Frozen Sections

Thymic lobes were removed from 14-to-16-day-old embryos embedded in Tissue-Tec on specimen stubbs, frozen on solid $CO_2$, and stored at −35°C. Six-micron frozen sections were cut on a Leitz cryostat and processed further for immunofluorescence as described (van Ewijk et al., 1988). ER-TR4 and ER-TR5 antibodies were used as primary antibodies, they were detected as described before.

### Fluorescence Microscopy and Flow Cytometry

A Zeiss Axiophot epifluorescence microscope equipped with LP615, FT600, 530-585, and LP520, FT510, 450-490 filters was used for detection and photography of Texas red and FITC-labeled cells, respectively. A FACScan instrument (Becton Dickinson) was used for immunofluorescence analysis of single-cell suspensions. Emitted fluorescence was detected with logarithmic amplification. Data collected from $1 \times 10^4$ cells were analyzed with Consort 30 software.

### ACKNOWLEDGMENTS

We thank Ane Marie Rulykke for expert technical assistance. This work was supported by the Danish Resarch Council and the C. L. David Foundation.

*(Received October 12, 1994)*

*(Accepted November 29, 1994)*

### REFERENCES

Berrih S., Arenzana-Selsoedos F., Cohen S., Devos R., Charron D., and Virelizler I.J. (1985). Interferon-gamma modulates HLA Class II antigen expression on cultured thymic epithelial cells. J. Immunol. **135**: 1165–1171.

Bhattacharya A., Dorf M.E., and Springer T.A. (1981). A shared alloantigenic determinant on Ia antigens encoded by the I-A and I-E subregions: Evidence for I region gene duplication. J. Immunol. **127**: 2488–2495.

Boyd R.L., and Hugo P. (1991). Towards an integrated view of thymopoiesis. Immunol. Today **12**: 71–79.

Cirne-Lima E.O., van Ewijk W., and Savino W. (1993). Cortical and medullary phenotypes within a mouse thymic epithelial cell line. In Vitro Cell. Dev. Biol. **29A**: 443–445.

Cordier A.C., and Haumont S.J. (1980). Development of thymus, parathyroid and ultimobranchial bodies in NMRI and nude mice. Amer. J. Anat. **157**: 227–263.

Cordier A.C., and Heremans J. F. (1975). Nude mouse embryo: Ectodermal nature of the primordial thymic defect. Scand. J. Immunol. **4**: 193–196.

Haynes, B.F., Scearce R.M., Lobach D.F., and Hensley L.L. (1984). Phenotypic characterization and ontogeny of mesodermal-derived and endocrine epithelial components of the human thymic microenvironment. J. Exp. Med. **159**: 1149–1168.

Hugo P., Boyd R.L., Waanders G.A., and Scollay R. (1991). CD41 + CD8 + CD3high thymocytes appear transiently during ontogeny: Evidence from phenotypic and functional studies. Eur. J. Immunol. **21**: 2655–2660.

Hugo P., Kappler J.W., Godfrey D.I., and Marrack P.C. (1992). A cell line that can induce thymocyte positive selection. Nature **360**: 679–682.

Hugo P., Kappler J.W., Godfrey D.I., and Marrack P.C. (1994). Thymic epithelial cell lines that mediate positive selection can also induce thymocyte clonal deletion. J. Immunol. **152**: 1022–1031.

Hugo P., Kappler J.W., McCormack J.E., and Marrack P.C. (1993). Fibroblasts can induce thymocyte positive selection in vivo. Proc. Natl. Acad. Sci. USA **90**: 10335–10339.

Lampert I.A., and Ritter M.A. (1988). The origin of the diverse epithelial cells of the thymus: Is there a common stem cell? In: The microenvironment of the human thymus, Kendall M. D. and Ritter M.A., Eds. (Chur: Harwood Academic Publishers), pp. 5–25.

Ozato K., Mayer N., and Sachs D.H. (1980). Hybridoma cell lines secreting monoclonal antibodies to mouse H-2 and Ia antigens. J. Immunol. **124**: 533–540.

Petersen O.W., and van Deurs B. (1988). Growth factor control of myoepithelial-cell differentiation in cultures of human mammary gland. Differentiation **39**: 197–215.

Ritter M.A., and Boyd R.L. (1993). Development in the thymus: It takes two to tango. Immunol. Today **14**: 462–469.

Röpke C., and Elbroend J. (1992). Human thymic epithelial cells in serum-free culture: Nature and effects on thymocyte cell lines. Develop. Immunol. **2**: 111–121.

Röpke C., Petersen O.W., and van Deurs B. (1990). Short-term cultivation of murine thymic epithelial cells in a growth factor defined serum-free medium. In Vitro·Cell. Dev. Biol. **26**: 671–681.

Shores E.W., van Ewijk W., and Singer A. (1991). Disorganization and restoration of thymic medullary epithelial cells in T cell receptor-negative scid mice: Evidence that receptor-bearing lymphocytes influence maturation of the thymic microenvironment. Eur. J. Immunol. **21**: 1657–1661.

Singer K.H., and Haynes B.F. (1987). Epithelial-thymocyte interactions in human thymus. Human Immunol. **20**: 127–144.

Van Ewijk W. (1991). T-cell differentiation is influenced by thymic microenvironments. Anu. Rev. Immunol. **9**: 591–615.

Van Ewijk W., Ron Y., Monaco J., Kappler J. Marrack P., Le Meur M., Gerlinger P., Durand B., Benoist C., and Mathis D. (1988). Compartmentalization of MHC class II gene expression in transgenic mice. Cell **53**: 357–370.

Van Ewijk W., Shores, E. W., and Singer A. (1994). Crosstalk in the mouse thymus. Immunol. Today **15**: 214–217.

Van Vliet E., Jenkinson E.J., Kingston R., Owen J.J.T., and Van Ewijk W. (1985). Stromal cell types in the developing thymus of the normal and nude mouse embryo. Eur. J. Immunol. **15**: 675–681.

Van Vliet E., Melis M., and Van Ewijk W. (1984). Monoclonal antibodies to stromal cell types of the mouse thymus. Eur. J. Immunol. **14**: 524–529.

Von Gaudecker B. (1991). Functional histology of the human thymus. Anat. Embryol. **183**: 1–15.

Vukmanovic S., Grandea III A.G., Faas S.J., Knowles B.B. and Bevan M.J. (1992). Positive selection of T-lymphocytes induced by intrathymic injection of a thymic epithelial cell line. Nature **359**: 729–732.

